# Editorial: The Role of Financing, Delivery, and Policy Innovations in Decreasing Chronic Disease Burdens

**DOI:** 10.3389/fpubh.2016.00237

**Published:** 2016-10-27

**Authors:** Joris van de Klundert, Steven W. Howard, Stephanie Bernell

**Affiliations:** ^1^Erasmus University Rotterdam, Rotterdam, Netherlands; ^2^Saint Louis University, St. Louis, MO, USA; ^3^Oregon State University, Corvallis, OR, USA

**Keywords:** chronic disease, financing, policy innovations, managed care programs, communicable diseases

**The Editorial on the Research Topic**

**The Role of Financing, Delivery, and Policy Innovations in Decreasing Chronic Disease Burdens**

Chronic conditions, such as high blood pressure and diabetes, are major contributing factors to global disease burdens (1). Next to clinical components, effective interventions for chronic diseases involve coordination and management of services, as well as the self-management and improved health behavior of patients. To date, a large portion of research has been devoted to clinical practices and local levels, such as an outpatient clinic or a primary care center. The resulting studies provide important insights in their specific contexts and disease episodes. However, they may fall short of generating evidence and theoretical insights on how interactions between various stakeholders (e.g., provider organizations, informal caregivers, and patients) jointly determine patient outcomes (2). This special issue presents a collection of articles aimed at advancing prevention and reduction of the burden of chronic disease, taking the network formed by the interacting stakeholders as the focus of analysis.

The contribution by Bernell and Howard points out that while chronic disease is widely recognized for its growing contribution to the burden on global health, both popular and scientific sources vary in their definition and use of the term “chronic disease.” For instance, ambiguities arise when considering the time period during which health is affected by chronic conditions, and when considering the extent to which conditions are curable, manageable, or fatal. Their review even points at an implicit debate around the issue of whether communicable diseases can be classified as chronic. Taking a practical and solution-oriented stance, they advocate to steer the definition away from etiological classifications and instead base it on observable disease burden.

Kelly et al. consider the improvement of service networks aiming to reduce obesity in New York City under the Bloomberg administration. Their qualitative study accurately shows the complexity of service delivery networks and the competing interests of supporting and opposing forces. These authors point out that the causes for obesity are not only complex but also contested. The case study presented by the authors confirms that strong leadership, in combination with operational capability, can effectively deliver improvement of public value, such as reducing risk factors for obesity.

In the USA, managed care is an approach which aims to improve the coordination in health service delivery networks and which appears to be well suited to improve outcomes for the chronically ill. Reasoning along these lines, Howard et al. hypothesize that managed care market penetration is associated with lower diabetes prevalence and lower prevalence of diabetes-related comorbidities. The presented analysis of Medicare Advantage, the private managed care option within the government’s Medicare program, does not support any significant effect of managed care market penetration on prevalence or incidence of diabetes and comorbidities. The findings raise questions about differences in service offerings and possible spill-over effects. It may also echo some of the difficulties and complexities of effective implementation of service network interventions encountered for obesity prevention in New York.

The struggle for effective provider-side interventions to improve outcomes for the chronically ill, partly stems from the importance of co-creation efforts of patients and informal caregivers, e.g., in the form of behavioral or lifestyle changes. Trivedi et al. do not start from the institutional context or provider-side interventions but instead develop a behavior change model to determine self-management barriers for patients suffering from heart failure. On the basis of this model and patient feedback, they subsequently designed, implemented, and evaluated the self-management program SUCCEED, which addresses couples of patients and significant others. The before–after analysis results on a sample of 17 couples shows encouraging results and poses important questions for attribution, especially regarding the effects on the caregivers, of which there are 50 million in the USA alone.

While chronic disease at present receives much attention for its growing share in the burden of disease in developed countries, it increasingly forms an important component of the burden of disease in developing countries as well. Many of these countries have difficulty freeing financial and human resources to meet health service demands, causing citizens to rely on co-payments, which may be especially demanding for chronic (continuous or reoccurring) conditions. Microfinancing systems have therefore received some attention in the literature as an instrument to reduce the burden of disease by improving health awareness and behavior. Dhungana et al. evaluate the impact of microfinancing pilots in four districts of Western Nepal, representing various ethnic groups. Their study provides evidence that the microfinancing interventions have been effective for improving awareness and behavior but that not all populations benefit equally. They find higher effectiveness among higher castes and suggest that future implementations should be adjusted to improve equity of effects.

The studies in this research topic underscore the global need to advance scientific understanding of network-level interventions to address the burden of disease stemming from chronic illness. Even the debate on definitions still requires careful attention. Together, the studies clearly illustrate that the improvement of service delivery for the chronically ill poses many multi-faceted challenges. Moreover, they elucidate the complexities of social interactions among a network of stakeholders involving industry, financers, government, health service providers, informal caregivers, and patients. These network interactions in turn relate in complex ways to patient outcomes, which are far from fully described or understood and warrant closer attention in the body of public health research.

## Author Contributions

All authors listed have made substantial, direct, and intellectual contribution to the work and approved it for publication.

## Conflict of Interest Statement

The authors declare that the research was conducted in the absence of any commercial or financial relationships that could be construed as a potential conflict of interest.
